# Cocoa Honey (*Theobroma cacao* L.): A Promising Byproduct for Industrial Applications

**DOI:** 10.1021/acsomega.6c01229

**Published:** 2026-05-14

**Authors:** Manuela B. Nascimento, Bruno N. Paulino, Joseane C. G. Alencar, Joselene C. N. Nascimento, Paulo R. R. Mesquita

**Affiliations:** † Center for Agricultural, Environmental and Biological Sciences, 186074 Federal University of Recôncavo da Bahia, Rua Rui Barbosa, 710, 44380-000 Cruz das Almas, BA, Brazil; ‡ Agricultural Technological Center of the State of Bahia (CETAB), Secretariat of Agriculture, Livestock, Irrigation, Fisheries and Aquaculture of the Government of the State of Bahia (SEAGRI), Ondina, 40170-110 Salvador, Bahia, Brazil; § Faculty of Pharmacy, Federal University of Bahia, Campus Ondina, 40170-115 Salvador, Bahia, Brazil

## Abstract

Cocoa honey is a sweet, acidic liquid obtained from the pulp of cocoa fruits (*Theobroma cacao* L.) during postharvest processing, distinct from bee honey and derived from cocoa pulp, being specifically defined as the liquid obtained by natural drainage of the pulp. Rich in soluble solids, total sugars, and essential minerals like magnesium and zinc, while low in sodium, its characteristic fruity, sweet, and floral aroma is attributed to high levels of esters. Despite its technological potential, the industrial exploitation of cocoa honey is hindered by significant research gaps, including a lack of standardized analytical methods, limited stability data, and insufficient economic assessment. This review provides a comprehensive analysis of the nutritional, physicochemical, and aromatic properties of cocoa honey, highlighting its innovative applications in the food, beverage, and cosmetic industries. Furthermore, we discuss the role of volatile organic compounds (VOCs) as potential biomarkers for quality control and prevention of food fraud. By addressing sustainability and the circular economy, this work identifies the critical path for transforming cocoa honey into a stable, safe, and commercially competitive ingredient, ultimately enhancing the cocoa value chain and the producer’s income.

## Graphical abstract

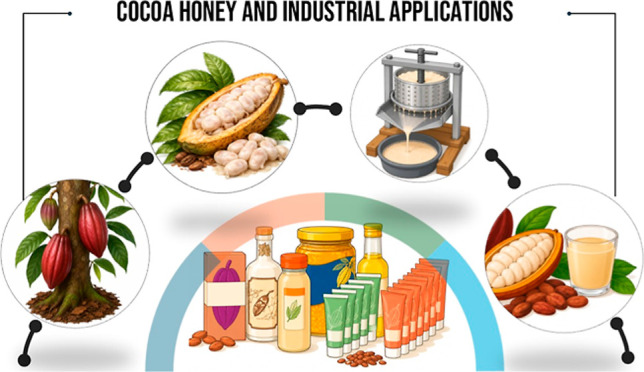

## 1 Introduction

Cocoa (*Theobroma cacao* L.), a tropical fruit highly valued worldwide, is primarily cultivated for its seeds, which are used to produce products such as chocolate, cocoa powder, and cocoa butter.^1^ It is a fruit from the Malvaceae family, which grows directly on the trunk and branches of the cocoa tree, which can reach up to 10 m in height, producing fruits up to 25 cm long.^2^ In 2024, global cocoa production reached around 4.5 million tons, with African countries contributing 71.2% of the total. The Ivory Coast and Ghana are the top producers, with 1.8 million and 580 thousand tons, respectively.^3^

However, cocoa production extends beyond final products. The fruit comprises the shell, beans, and pulp, which constitute approximately 70%, 20%, and 10% of the entire fruit, respectively. This represents a substantial volume of biomass, much of which is underutilized or discarded, contributing to environmental and waste-management challenges.^1,4,5^ Previous reviews have primarily emphasized nutritional composition or broad sustainability aspects. However, little attention has been given specifically to cocoa honey, particularly regarding the integration of its chemical composition, the volatile organic compound (VOC) profile in relation to different cocoa varieties, preservation technologies, and authenticity markers. Furthermore, the industrial translation of cocoa honey, including challenges related to processing, stability, scalability, and regulatory acceptance, remains underexplored in the literature.^5,6^

In this regard, agricultural byproducts represent a valuable source of raw materials for the industry, with the potential to yield high-value-added products. In this context, the industry has invested in innovative solutions to transform these byproducts into new products or inputs. The pursuit of sustainable alternatives is crucial to ensuring the long-term viability of cocoa production, balancing economic development with environmental preservation.^7,8^

Cocoa honey is a yellowish liquid that exudes from cocoa beans prior to fermentation and is characterized by its sweet flavor and rich composition of sugars, organic acids, vitamins, and minerals. It is specifically defined as the liquid obtained by natural drainage of the pulp (sweatings), distinguishing it from closely related matrices such as cocoa mucilage (the pulp adhering to the beans) and cocoa pulp juice obtained by a mechanical pulper. However, in industrial and small-scale processing, liquids obtained by mechanical pressing using stainless-steel presses are sometimes also referred to as cocoa honey.^9,10^ In this review, these related matrices are considered only when directly relevant for compositional comparison or technological discussion. During the processing of cocoa beans for chocolate production, cocoa honey remains underutilized, highlighting the need for comprehensive studies to optimize its use and explore its potential applications in the food and beverage industry.^9,11^ However, because of its high sugar and moisture content, cocoa honey has a short shelf life and is prone to fermentation, hindering its commercialization. Therefore, developing preservation methods and exploring its incorporation into food products are essential to ensure quality, stability, and market potential.^6,9,12^

Sustainable cocoa processing innovations have increasingly focused on circular economy approaches and zero-waste strategies, aiming to reduce waste and add value to byproducts from the cocoa production chain. These approaches create new opportunities for producers, particularly in developing countries, by promoting resource efficiency and diversification of products.^8,13^ Recent studies have highlighted the functional and technological potential of cocoa byproducts, including cocoa husk, cocoa pulp, and cocoa bean shell; however, information on cocoa honey remains limited. In this context, cocoa honey emerges as a promising yet underexplored byproduct within zero-waste cocoa processing systems.^5^ This review aims to explore current trends in cocoa honey, deepen scientific understanding of this matrix, and highlight its potential to drive innovation and sustainability in the cocoa industry.

## 2 Review Methodology

This Review was conducted based on a structured literature search to ensure transparency and reproducibility. Scientific articles were retrieved from major databases, including Scopus, Web of Science, and Google Scholar, covering publications from April 2014 to March 2025 (the last search performed on April 3, 2026).

The search strategy combined keywords using Boolean operators (AND, OR), including: (“cocoa honey” OR “cocoa pulp exudate” OR “cocoa mucilage”) AND (“*T. cacao*”) AND (“composition” OR “volatile organic compounds” OR “VOCs” OR “processing” OR “preservation” OR “spray drying”). Database-specific adaptations of the search strings were applied where necessary.

The selection process was conducted in successive stages, including screening of titles and abstracts, followed by full-text assessment of potentially relevant studies. Studies were selected based on their relevance to the chemical composition, volatile profile, processing, preservation, and technological applications of cocoa honey. Only peer-reviewed articles written in English were included, while studies not directly related to cocoa-derived products or lacking sufficient methodological details were excluded.

Duplicate records were identified and removed manually during the screening process. In addition, backward citation searching (screening the reference lists of selected articles) was performed to ensure comprehensive coverage of relevant literature.

In addition, patent data were collected to assess technological developments related to cocoa honey processing and utilization. Patent searches were conducted using Espacenet and Google Patents. The search terms included (“cocoa pulp” OR “cocoa honey” OR “cocoa mucilage”) AND (“processing” OR “extraction”). Relevant patents were selected based on their applicability to industrial processes, preservation techniques, or product development involving cocoa honey.

## 3 Process of Obtaining Cocoa Honey

The process of obtaining cocoa honey begins with breaking the pods and removing the fresh cocoa beans, which are surrounded by a mucilaginous pulp. In this review, cocoa honey is defined as the liquid obtained by gravity drainage, also known as “sweatings,” in which the pulp naturally drains from the beans without the application of external force. However, it is important to note that in industrial and small-scale production contexts, liquids obtained by mechanical pressing are sometimes also referred to as cocoa honey, although they may differ in composition and extraction characteristics. Mechanical pressing involves the application of external pressure to extract liquid from the pulp, typically resulting in higher yields. The extracted liquid, known as cocoa honey, corresponds to approximately 5–6% of the fresh fruit’s weight (Figure 1). Traditional wooden presses are often used during cocoa pulp extraction. However, wood is a porous material that can absorb moisture and organic residues, making it more difficult to properly clean and sanitize compared to nonporous food-grade materials such as stainless steel. Consequently, when not properly treated or maintained, wooden equipment may increase the risk of microbial contamination. According to the Codex Alimentarius Commission General Principles of Food Hygiene (2020), surfaces that come into contact with food should be made of materials that are smooth, nonabsorbent, and easy to clean and sanitize, characteristics that help reduce the risk of contamination.^4,6,14^

**Figure 1:**
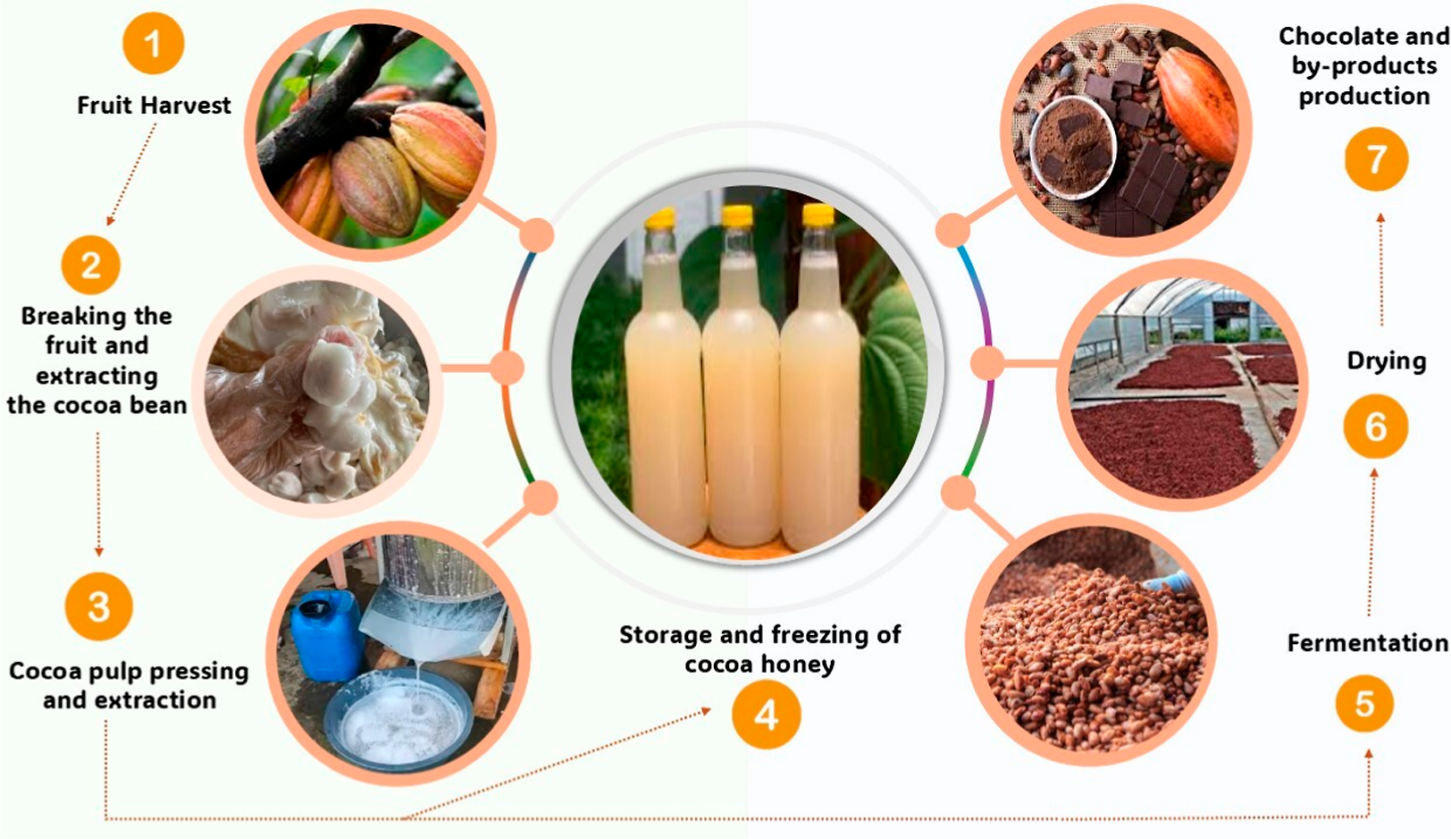
Cocoa honey extraction process (*Theobroma cacao* L.).

In addition to these conventional methods, recent studies have reported alternative extraction approaches, including the use of hydraulic presses, screw presses, and controlled fermentation systems designed to enhance the pulp separation and liquid recovery.^15,16^ These methods aim to improve the process efficiency, standardization, and scalability for industrial applications.

Therefore, the safe processing of cocoa honey involves the selection of healthy cocoa pods, proper sanitization, and the use of stainless-steel presses under hygienic conditions. Following extraction, the liquid should be filtered to remove impurities and subjected to heat treatment, such as pasteurization (e.g., 65 °C/30 min), or to emerging technologies, including ultrasound (e.g., 20 kHz/10 min) or high-pressure processing (e.g., 300 MPa and 4 °C), to reduce the microbial load. Cocoa honey is naturally acidic, featuring a pH between 3.56 and 3.69. This acidity contributes to inhibiting pathogen growth and increases the effectiveness of heat treatments. Subsequently, the cocoa honey should be rapidly cooled, bottled in sterilized containers, hermetically sealed, and stored under refrigeration to ensure both the quality and safety of the final product.^17,18^ Furthermore, membrane-based technologies and enzymatic treatments have also been explored to improve clarification, filtration efficiency, and stability of cocoa pulp-derived products, representing promising alternatives for industrial-scale processing.^19,20^

The yield of cocoa honey, based on the quantity of fresh cocoa beans, varies according to the cocoa variety used.^21^ According to the research, the varieties CCN51, SJ02, and PS1319 exhibited the highest yields (20.44%, 18.04%, and 17.97%, respectively), compared to the Parazinho variety (16.40%), considering the fresh pulp fraction. The yield determinations were performed in triplicate. This result can be attributed to the fact that CCN51, SJ02, and PS1319 are genetically improved Trinitário cocoa varieties, which have a higher pulp content, thus promoting greater productivity. However, differences in yield may also be influenced by other factors, such as agronomic conditions, fruit maturity stage, pod size, pulp adhesion, and postharvest handling. In contrast, the Parazinho variety, which belongs to the Forastero group, is characterized by a lower pulp content, resulting in a reduced yield.

After the removal of cocoa honey (Figure 1), the beans proceed to the fermentation stage in troughs, during which flavor and aroma precursors are formed. They then undergo drying and roasting, after which the roasted beans are cracked to remove the shells and obtain the cocoa nibs. The nibs are subsequently ground, releasing cocoa butter, and forming cocoa mass, the base for chocolate and other derivatives.^22–24^

Research has demonstrated that removing up to 60% of cocoa pulp does not significantly alter the sensory properties of chocolate when conducted under controlled processing conditions, such as appropriate fermentation time and temperature, cocoa cultivar, and drying regime, which are key factors influencing fermentation dynamics and final product quality.^5,25^ These findings indicate that cocoa honey can be extracted as a value-added byproduct without compromising chocolate quality under the specific cultivars, controlled fermentation, and drying conditions evaluated and should not be generalized beyond these conditions. Nevertheless, beyond sensory attributes, factors such as fermentation kinetics, acidification, and microbial ecology should also be considered when assessing the effects of the pulp removal. While this practice may not substantially impact fermentation under controlled conditions, it can reduce substrate availability for yeasts, lactic acid bacteria (LAB), and acetic acid bacteria (AAB), thereby potentially affecting microbial activity, heat generation, acid production, and overall bean quality.^25^ Haruna et al.^26^ also reported the benefits of partial pulp removal in cocoa beans from Ghana.

## 4 Stability and Preservation Methods

Fresh cocoa honey is rapidly fermented due to its high content of reducing sugars, such as fructose and glucose, as well as its high moisture content, which limits its shelf life. Currently, cocoa honey is primarily marketed in a frozen form; however, logistical challenges hinder its widespread distribution and export.^27^

Studies suggest the application of various preservation methods to extend the shelf life of cocoa honey. An analysis of the effects of thermal pasteurization (65 °C/30 min), thermal pasteurization with additives (65 °C/30 min and 0.1 *g*/100 mL potassium sorbate), and high-intensity ultrasound (20 kHz/10 min) on the storage of cocoa honey at 4 °C for 28 days revealed that pasteurization with additives and ultrasound were the most effective treatments, preserving cocoa honey for 28 and 21 days, respectively.^17^

The effects of thermal pasteurization (TP) (65 °C for 30 min) and high-pressure processing (HPP) (500 MPa for 4 min and 600 MPa for 3 min) on the quality attributes of cocoa honey were evaluated during 21 days of refrigerated storage (4 °C). The results showed that HPP at 600 MPa for 3 min achieved a 5-log reduction of *Escherichia coli* O157:H7 and completely inhibited microbial growth throughout the storage period. Additionally, cocoa honey treated at 500 MPa for 4 min exhibited the highest antioxidant capacity. Overall, HPP did not significantly compromise the quality attributes of cocoa honey and achieved effects comparable to thermal pasteurization, while providing enhanced microbial stability during storage. These findings highlight the potential of HPP as an alternative preservation strategy, supporting the production of high-quality cocoa honey and offering new perspectives for storage methods aligned with both consumer and industrial demands.^28^

The spray-drying method presents a viable alternative for the large-scale stabilization of products such as cocoa honey, as it offers reduced exposure to heat compared to conventional drying methods. The study by Guirlanda et al.^27^ addressed the development of a drying technology to stabilize cocoa honey in powder form using atomization, also known as spray drying. The process was conducted with an inlet temperature of 180 °C and an outlet temperature of 85 ± 3 °C, using a controlled feed rate to ensure drying efficiency and the retention of thermosensitive compounds. Combinations of maltodextrin, methocel, and whey protein isolate (WPI) were tested. The study concluded that the formulation containing maltodextrin and whey protein isolate, at a ratio of 29:1, resulted in a more stable product, with the lowest loss of phenolic compounds during the drying process (6.04%). These results are promising for extending the shelf life of cocoa honey, thereby opening new opportunities for its commercialization. Although the use of WPI improves stability and nutritional value, its industrial application requires attention to mandatory labeling, as whey proteins are known allergens. In Brazil, regulations such as RDC 727/2022 must be considered, since cow’s milk protein allergy is one of the most common food allergies.^29^

The thermal stability of cocoa honey indicates that temperatures above 75.9 °C trigger irreversible molecular changes and mass losses between 20% and 30%, with this threshold corresponding to the onset of thermal degradation identified under analytical conditions, rather than a directly applicable processing limit. Although the range of 75–80 °C is commonly used in commercial food pasteurization, these data should not be directly extrapolated to process design without validation in the specific cocoa honey matrix. Process optimization for this product should integrate parameters such as time, temperature, and pH to ensure pathogen elimination while maintaining a safety margin, without causing irreversible changes in the molecular structure or excessive loss of antioxidants. This approach is recommended as a strategy to transform cocoa honey into a viable industrial ingredient.^12^ While this strategy is technically feasible, further validation under industrial processing conditions is required to ensure consistent product safety and quality.

In the study by Haase et al.,^30^ pasteurization (80 °C for 30 s) and ultrahigh temperature (UHT) treatment (135 °C for 30 s) were shown to be effective technologies for cocoa pulp preservation, as they successfully inactivated microorganisms and enzymes. However, UHT treatment combined with refrigerated storage at 4 °C for up to 24 weeks proved to be the most effective approach for maintaining product stability over time. Although this review focuses on cocoa honey, the study addresses cocoa pulp, a similar product, as its extraction also results in a liquid rich in soluble compounds, allowing relevant inferences regarding preservation strategies.

Overall, while pasteurization and ultrasound show potential for short-term stabilization and spray drying has been widely explored as a promising approach for enabling long-distance commercialization, other technologies such as ultrahigh-temperature (UHT) treatment and high-pressure processing (HPP) have also demonstrated strong potential for ensuring microbial safety and extending shelf life while preserving quality attributes. Nonetheless, comparative studies addressing economic feasibility, scalability, and consumer acceptance across these preservation strategies are still lacking and are essential to support their industrial adoption.

## 5 Nutritional Composition

Cocoa honey has a diverse composition, being rich in sugars, organic acids, vitamins, minerals, bioactive compounds, and dietary fibers. Its antioxidant properties, along with its nutritional and physicochemical characteristics, make it a promising ingredient for various applications in the food industry.^9,11^ Table 1 presents the physicochemical and nutritional composition of cocoa honey, as reported in different studies.

**Table 1:** Physicochemical and Nutritional Composition of Cocoa Honey (*Theobroma cacao* L.)

composition	amount	reference
*Physicochemical*		
pH	2.76–3.77	^17,21,28,31,32^
total acidity (% citric acid)	0.68–1.04	^17,21,32^
moisture (%)	69.44–87.22	^17,31,32^
solid soluble total (°Brix)	11.97–17.32	^17,28,31,32^
ash (%)	0.23–0.59	^17,31,32^
protein (%)	0.31–1.20	^17,31^
carbohydrates (%)	11.80–29.00	^17,31^
pectin (%)	0.36–1.50	^2,31^
fat (%)	0.05–0.19	^12^
total fiber (%)	0.63	^12^
soluble food fiber (%)	0.25	^12^
insoluble food fiber (%)	0.37	^12^
turbidity (NTU)	421.67	^12^
conductivity (mS cm^–1^)	2.72	^12^
energy value (kcal/100 mL)	113.58–120.53	^17^
energy (kJ/100 mL)	477.03–506.23	^17^
*Organic acids*		
malic acid (mg L^–1^)	3.6	^2^
lactic acid (mg L^–1^)	1.23	^2^
oxalic acid (mg L^–1^)	1.27	^2^
citric acid (mg L^–1^)	9.14	^2^
acetic acid (mg L^–1^)	2.28	^2^
*Color parameter*		
L*	93.103–99.579	^17^
a*	–0.069–2.02	^17,30^
b*	8.010–12.912	^17,30^
C*	8.024–12.943	^17^
h°	86.146–93.108	^17^
*Sugar*		
sucrose (g 100 mL^–1^)	7.31–8.32	^12^
glucose (g 100 mL^–1^)	4.19–4.58	^12,31^
fructose (g 100 mL^–1^)	3.25–4.82	^12,31^
reducing sugars (g 100 mL^–1^)	5.03–9.01	^17^
nonreducing sugars (g 100 mL^–1^)	4.29–10.20	^17^
total sugars (g 100 mL^–1^)	12.29–16.96	^17^
*Vitamin*		
vitamin B3 [nicotinic acid] (mg 100 mL^–1^)	0.67	^12^
vitamin B5 [calcium pantothenate] (mg 100 mL^–1^)	0.28	^12^
vitamin B7 [biotin] (mg 100 mL^–1^)	0.16	^12^
*Mineral*		
Ca (mg L^–1^)	56.0–159.0	^11^
K (mg L^–1^)	734.0–1002.0	^11^
P (mg L^–1^)	16.9–65.4	^11^
Mg (mg L^–1^)	54.2–78.0	^11^
Fe (mg L^–1^)	1.12–3.58	^17,31^
Na (mg L^–1^)	46.16–97.97	^17^
Zn (mg L^–1^)	0.40–19.44	^17,31^

In the analysis of the physicochemical composition of cocoa honey from the varieties CCN51, PS1319, SJ02, and Parazinho, the product was characterized as acidic, with a pH ranging from 2.76 to 3.77. It exhibited a high sugar content (12.29–16.96 g 100 mL^–1^), total soluble solids (11.97–17.32 °Brix), and carbohydrates (11.80–29.00%), resulting in a considerable energy value. The high sugar concentration relative to acidity imparts a sweet sensory profile, making cocoa honey an attractive ingredient for various food formulations. Furthermore, colorimetric analysis revealed high luminosity, with L values close to 100. It was also observed that the PS1319 and SJ02 varieties exhibited similar color profiles, tending toward a slightly brownish hue, while the CCN51 and Parazinho varieties displayed a slightly greenish hue, positively contributing to the visual appeal of the product and expanding its potential applications in the food industry. Considering the Recommended Daily Intake (RDI) of 7 mg for zinc recommended by Brazilian legislation, the consumption of cocoa honey from the CCN51 and PS1319 varieties highlights its contribution to zinc intake, rather than characterizing it as a significant source of this mineral. Therefore, the consumption of cocoa honey, either directly or as an ingredient in food products, may contribute to improving the nutritional profile of foods due to the presence of nutrients and bioactive compounds. However, its use should be considered in the context of a balanced diet, particularly given its natural sugar content.^21^

Cocoa honey has a low lipid content (0.05–0.19%) and contains total dietary fiber (0.63%), distributed between soluble (0.25%) and insoluble (0.37%) fractions.^12^ Although the fiber content is relatively low, it contributes to the overall nutritional composition of cocoa honey and may support digestive health when consumed as part of a balanced diet.^33^ Although studies specifically addressing the composition of cocoa honey remain limited, additional insights can be drawn from the cocoa exudate, the precursor liquid from which it is derived. Cocoa exudate is characterized by a low pH and a sour sensory profile, largely attributed to the presence of organic acids such as citric, malic, lactic, acetic, and oxalic acids, which may also be present in cocoa honey, depending on processing conditions. Reported concentrations include citric acid (9.14 mg L^–1^), malic acid (3.6 mg L^–1^), acetic acid (2.28 mg L^–1^), oxalic acid (1.27 mg L^–1^), and lactic acid (1.23 mg L^–1^), as reported in the original study, although these values appear low relative to the total acidity and may reflect differences in analytical methods or reporting basis. It is important to emphasize that these compounds play an important role not only in sensory perception but also in microbial stability and fermentation dynamics. In addition, the cocoa exudate contains pectic substances, with reported values ranging from 0.57 to 1.5%, which may be partially transferred to cocoa honey during extraction, contributing to its viscosity and technological properties. However, direct data on the pectin content in cocoa honey are still scarce, highlighting an important research gap.^2^

In this context, cocoa honey stands out as a nutritious and versatile food ingredient serving as a natural source of several nutrients and bioactive compounds. However, further studies, particularly in humans, are needed to better understand their potential health effects. Additionally, its composition may vary not only according to the cocoa variety but also due to seasonal factors, harvest period, and postharvest handling conditions, highlighting the need for standardized studies to more accurately characterize its physicochemical and functional properties.

## 6 Bioactive Components

Bioactive components play an important role in determining the nutritional, functional, and sensory properties of the foods. In cocoa honey, these compounds include both nonvolatile constituents, such as phenolic compounds, sugars, organic acids, and vitamins, as well as volatile organic compounds responsible for aroma and flavor characteristics. While nonvolatile compounds are mainly associated with the nutritional value and potential functional properties of the product, volatile compounds contribute to its sensory profile and may also serve as quality and authenticity markers. Therefore, the characterization of both groups of compounds is essential for a comprehensive understanding of the chemical composition and quality attributes of cocoa honey.^12,21,24^

Volatile organic compounds (VOCs) are essential for assessing food quality and influencing aroma and flavor. In cocoa honey, VOCs can serve as quality markers, indicating aspects such as origin, processing, and manufacturing practices. Gas chromatography coupled with mass spectrometry (GC–MS) is commonly used to detect and characterize VOCs in cocoa honey. However, the quantification of these compounds depends on the use of appropriate calibration and analytical standards, and many studies report semiquantitative data based on relative abundances.^12,34^ Recent studies have identified VOCs that highlight the primary quality markers of cocoa honey (Table 2).

**Table 2:** Volatile Organic Compounds (VOCs): Potential Markers of Cocoa Honey Quality (*Theobroma cacao* L.)

compound	odor descriptors	relative percentage %^a^	reference
*Alcohols*			
2-pentanol	alcoholic, fruity, citrus	2.23–10.47	^12,34^
2-heptanol	citrus, fresh, lemon-grasslike, sweet	0.05–2.06	^12,34^
2-nonanol	fruity	0.11–2.26	^34^
*Esters*			
2-pentyl acetate	fruity, herbal	54.06	^12^
1-methylbutylacetate	fruity	11.92–23.87	^34^
2-heptyl acetate	fruity	1.24–10.89	^34^
α-methylbenzyl acetate	sweet, fruity	1.19–6.87	^34^
2-nonyl acetate	fruity, sweet	5.88	^12^
*Ketones*			
2-heptanone	fruity, flowery, pear, grape	0.37–0.97	^34^
acetophenone	sweet, almond, flowery	0.01–2.00	^17,34^
2-nonanone	flowery, fruit	0.30–1.75	^34^
*Terpenes*			
d-limonene	citrus, orange, sweet	0.01–0.12	^17,34^
linalool	flowery, lavender, rose	0.13–2.76	^17,30,34^
nerol	floral, rose, fruity	0.49–0.78	^34^
(Z)-linalool oxide	sweet, nutty	0.22–1.04	^34^
(E)-linalool oxide	floral	0.45–1.64	^34^

a Relative percentage (%) calculated by GC–MS peak area normalization.

Cocoa honey contains a wide variety of compounds, which are classified into several chemical classes, including alcohols, esters, ketones, and terpenes. A study on volatile organic compounds (VOCs) and the sensory profile of cocoa honey from different varieties (CCN51, PS1319, SJ02, and Parazinho) using gas chromatography–mass spectrometry (GC–MS) was reported by Nascimento et al.^34^ This study identified 84 compounds, highlighting the presence of several substances responsible for the fruity, floral, citrus, and sweet aromas of cocoa honey. Among the compounds identified, the following are noteworthy: 2-pentanol (2.23–4.12%), 2-heptanol (0.05–2.06%), 1-methylbutyl acetate (11.92–23.87%), 2-heptyl acetate (1.24–10.89%), α-methylbenzyl acetate (1.19–6.87%), linalool (0.78–2.76%), and d-limonene (0.04–0.12%). Furthermore, the sensory profile was assessed with 20 trained tasters using ranking descriptive analysis (RDA), a sensory method in which panelists rank samples according to the intensity of specific attributes, allowing for comparative profiling without requiring absolute scaling. The association between VOCs and sensory attributes was explored using multivariate statistical analysis (e.g., principal component analysis), enabling the identification of relationships between chemical compounds and perceived aromas. The results revealed that the CCN51 variety was rich in acids, esters, and terpenes, exhibiting sensory attributes such as acidic, fruity, cocoa/cocoa pulp, and mint/refreshing aromas.

The analysis of volatile organic compounds (VOCs) in cocoa honey from the CCN51 variety, conducted by Rocha et al.,^12^ using gas chromatography–mass spectrometry (GC–MS), identified nine key substances, including alcohols and esters, which are responsible for the characteristic fruity and floral aroma of cocoa honey. Similar results were observed in the cocoa pulp, with the identification of compounds such as 2-pentanol, 2-heptyl acetate, 2-pentyl acetate, 2-nonanone, and linalool.^35^

Thus, the analysis of VOCs in cocoa honey represents a promising approach for monitoring the product quality and exploring potential authenticity markers. However, the practical application of these compounds for fraud detection and adulteration prevention requires further validation, including the establishment of reference ranges, standardized analytical protocols, and external validation data sets. From a market perspective, the diversity among cocoa varieties offers significant opportunities for product differentiation and value addition. For example, the CCN51 variety, characterized by fruity, floral, and refreshing notes, has the potential to appeal to consumers who seek exotic or gourmet products. Moreover, the identification of specific VOCs as potential quality markers supports the development of branding strategies and the establishment of geographical indications or origin labels, which are increasingly valued in international markets. Nevertheless, important challenges remain, including the need to expand analytical methodologies for routine quality control and to effectively communicate sensory diversity to consumers in a clear and accessible manner.

There is limited data in the literature regarding the nonvolatile compounds of cocoa honey, particularly its bioactive components. Molecular analysis conducted by Rocha et al.^12^ using nuclear magnetic resonance (NMR) identified amino acids at relatively low concentrations (sub-mg to mg per 100 mL), including threonine (0.4469 mg 100 mL^–1^), glycine (0.1107 mg 100 mL^–1^), alanine (4.9036 mg 100 mL^–1^), and arginine (6.6161 mg 100 mL^–1^). While amino acids are essential for physiological functions such as protein synthesis and tissue repair, the levels reported in cocoa honey are low, and their direct nutritional contribution is likely limited in typical consumption scenarios. Nonetheless, their presence may be relevant from a compositional and technological perspective.^36,37^

Regarding total phenolic compounds, Guirlanda et al.^27^ reported levels of 424.3 mg GAE g^–1^ in dried cocoa honey, using the Folin–Ciocalteu spectrophotometric method, while Rocha et al.^12^ found concentrations of 251.34 mg GAE 100 mL^–1^ in liquid cocoa honey. Due to differences in the sample matrix and expression basis, these values are not directly comparable but collectively indicate the presence of phenolic compounds in cocoa honey and its derivatives. It is well established that phenolic compounds contribute significantly to antioxidant activity through mechanisms such as free radical scavenging, metal chelation, and inhibition of oxidative processes.^38,39^ In addition, phenolic compounds have been associated with antimicrobial activity, as they can disrupt microbial cell membranes and interfere with enzymatic systems. However, it is important to note that the presence of reducing sugars in cocoa honey may interfere with the Folin–Ciocalteu method, potentially leading to an overestimation of phenolic content.^40^ Therefore, further studies employing more selective analytical techniques, such as liquid chromatography, are necessary for accurate identification and quantification of individual phenolic compounds.

Cocoa pulp is composed, on average, of high levels of sugars, which may reach approximately 76% of the soluble solid fraction, with a predominance of reducing sugars such as fructose and glucose, particularly in ripe fruits.^41^ Recently, for the first time, the naturally occurring sugar concentrations in cocoa honey were characterized, revealing high levels of sucrose (7.31 g 100 mL^–1^), glucose (4.19 g 100 mL^–1^), and fructose (4.65 g 100 mL^–1^) (Table 1). Fructose, in particular, has metabolic properties associated with a lower glycemic index compared with glucose. However, cocoa honey also contains significant amounts of glucose and sucrose and should be considered a free sugar-rich ingredient. Therefore, while its sugar profile may offer technological and sensory advantages, its use as an alternative to commercial sugar should be interpreted with caution and supported by further metabolic and clinical studies.^12^

B vitamins, including B3 (0.67 mg 100 mL^–1^), B5 (0.28 mg 100 mL^–1^), and B7 (0.16 mg 100 mL^–1^), are water-soluble and have been identified in cocoa honey from the CCN51 variety (Table 1). These vitamins play essential roles in bodily functions, acting as enzymatic cofactors, antioxidants, and contributing to hormone synthesis. However, given the concentrations reported, their contribution to daily nutritional requirements is likely limited and should be considered within the context of the overall dietary intake. Although some B vitamins can be synthesized endogenously in limited amounts, dietary intake is necessary to meet physiological requirements and maintain normal metabolic functions. Therefore, cocoa honey may be considered a source of these nutrients when consumed as part of a balanced diet.^42,43^

In this context, there is a growing interest in the bioactive properties of cocoa honey, particularly regarding its potential antioxidant and anti-inflammatory compounds, which opens avenues for the development of novel products, including functional ingredients and applications in the pharmaceutical sector. However, despite this increasing research interest, available data remain limited, especially concerning the detailed characterization of nonvolatile compounds and their biological effects.

## 7 Technological and Innovation Potential

Cocoa honey is an innovative product that has garnered attention not only for its unique flavor but also for its technological and innovative potential. Although its commercialization remains limited, cocoa honey is increasingly recognized because of the rising demand for natural, sustainable, and differentiated products in the market.^5,6,12^

To improve clarity, evidence derived directly from cocoa honey is distinguished from that obtained from related matrices (cocoa pulp, pulp juice, and mucilage), as summarized in Table 3.

**Table 3:** Summary of Evidence Derived Directly Cocoa Honey and from Related Matrices (Cocoa Pulp, Pulp Juice, and Mucilage), Highlighting Their Respective Applications^a^

evidence type	matrix	application/process	key findings	reference
direct	cocoa honey	fermentation	naturally rich in fermentable sugars, enabling microbial activity	^9^
direct	cocoa honey	beverages	high sugar content and pleasant sensory profile support its use in beverage formulations	^44^
direct	cocoa honey/cocoa pulp	fruit wine fermentation	use of Saccharomyces cerevisiae strains demonstrated the feasibility of producing fruit wines from cocoa honey supplemented with pulp, supporting yeast fermentation and product development	^45^
analogous	cocoa mucilage	fermented beverage (kombucha-like)	moderate mucilage levels improve sensory acceptance; viable substrate for fermentation	^46^
analogous	cocoa pulp juice	probiotic formulations	supports probiotic viability and release of bioactive compounds	^47^
analogous	cocoa pulp juice + husk	chocolate formulation	partial sugar replacement; increased fiber and reduced saturated fat under specific conditions	^48^
analogous	cocoa pulp juice	functional ice cream	maintains viability of Lactobacillus casei; enhances bioactive compounds and antioxidant activity; moderate sensory acceptance	^49^
direct (patent-based)	cocoa honey	fermented beverages (brandy)	describes production processes; represents claimed methods without independent validation	^50^
direct (patent-based)	cocoa honey	craft beer	used as a fermentable sugar source; associated with sensory and compositional attributes	^51^
direct (patent-based)	cocoa honey/cocoa pulp	chocolate (sugar substitute)	proposed as a natural sweetener; potential impact on flavor	^52^
direct (patent-based)	cocoa honey	cosmetic formulations	preliminary evidence suggests no irritation; limited methodological detail	^53^
direct (patent-based)	cocoa honey	fragrance industry	source of aromatic compounds; based on proposed applications in patents	^54^

a Analogous evidence should be interpreted with caution due to differences in extraction processes and composition.

The main applications of cocoa pulp byproducts include the production of alcoholic beverages (e.g., wine), functional beverages, such as kombucha, as well as juices, jams, and liqueurs (Figure 2), which are often produced through artisanal or semi-industrial processes.^5,6,44^ Direct evidence from cocoa honey-based systems, including formulations supplemented with a cocoa pulp, indicates its suitability for alcoholic fermentation. Studies on wine production from cocoa honey and cocoa pulp have shown that its natural sugar content and acidity provide suitable conditions for yeast fermentation, resulting in beverages with desirable sensory attributes such as fruity aroma and balanced acidity.^45^

**Figure 2:**
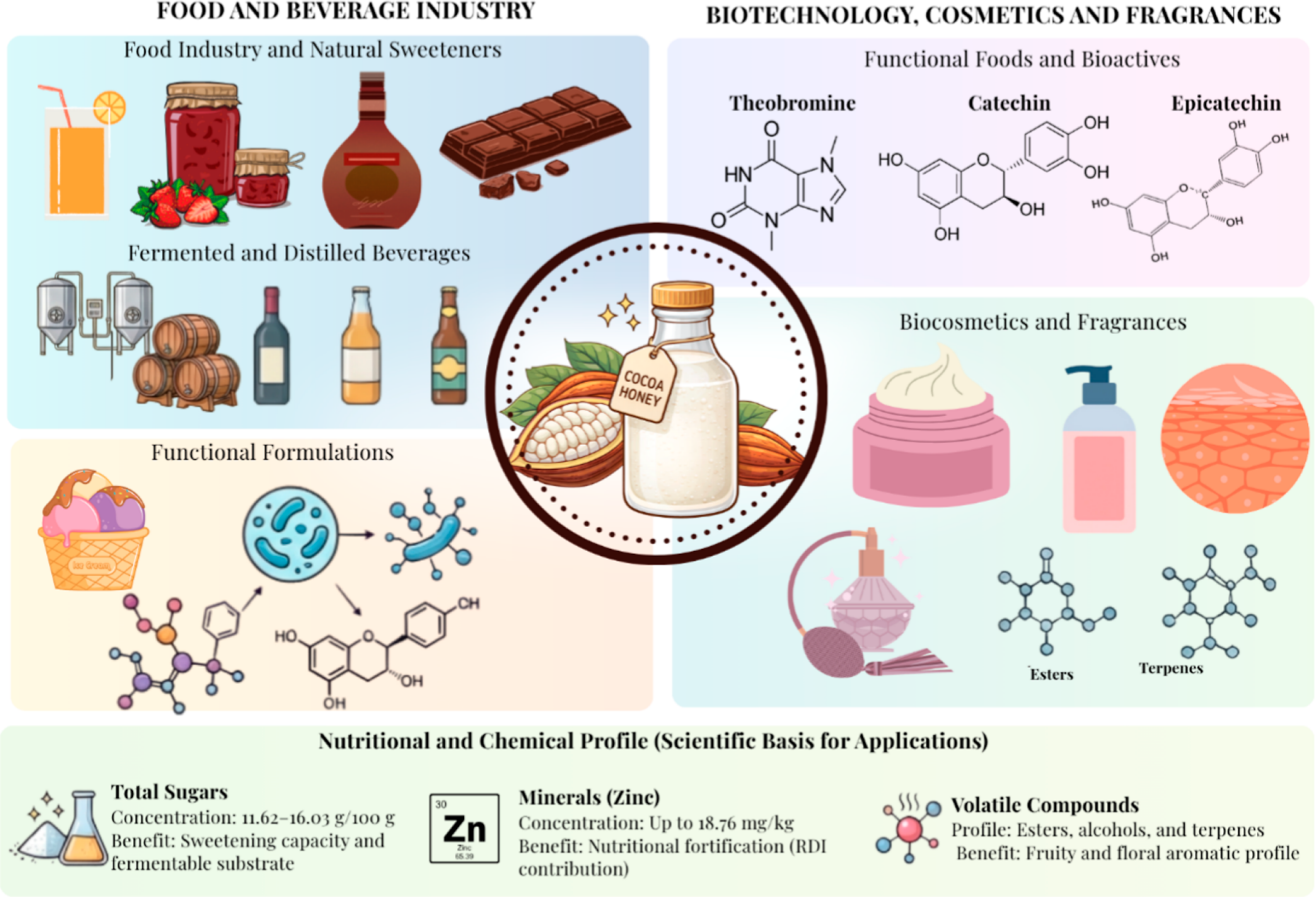
Technological applications of cocoa honey (*Theobroma cacao* L.). The listed products, including beverages, natural sweeteners, and cosmetic formulations, represent reported and potential applications of cocoa honey.

Direct evidence from cocoa honey indicates its suitability as a substrate for kombucha-type beverages. Cocoa honey has been investigated as an alternative substrate for symbiotic cultures of bacteria and yeasts (SCOBY), contributing to fermentation, the formation of organic acids, and the generation of bioactive compounds. After 6 days of fermentation, high antioxidant activity was observed (70.14% ± 1.15% DPPH and 43.49% ± 0.52% ABTS), along with significant concentrations of phenolic compounds (144.75 ± 2.03 mg GAEq/mL) and flavonoids (1.776 mg QAEq/mL).^9^ Analogous evidence from cocoa mucilage indicates the feasibility of developing kombucha-type fermented beverages. Rodríguez-Castro et al.^46^ evaluated two cocoa varieties, Nacional Cocoa Fino de Aroma (NCFA) and Colección Castro Naranjal 51 (CCN51). Using mature raw material, a prototype fermented beverage similar to kombucha was developed with different mucilage concentrations (40, 60, 80, and 100 g L^–1^). Among the sensory panelists, the CN40 treatment (Nacional mucilage +40 g L^–1^ sugar) showed the highest acceptability and was considered the most promising in terms of overall preference.

Furthermore, the production of juices and jams from the cocoa pulp has been widely reported, highlighting its high content of sugars, organic acids, and pectic substances, which contribute to gel formation, flavor, and overall product stability.^24^ Analogous evidence from cocoa pulp juice suggests the potential for the development of alcoholic beverages from cocoa-derived matrices. Liqueurs derived from cocoa pulp juice have also been explored, typically involving maceration and fermentation steps that enhance the extraction of the aromatic compounds. Despite the predominance of artisanal-scale production, these studies demonstrate the technological feasibility and versatility of cocoa pulp byproducts, reinforcing their potential for industrial applications and the development of value-added products.^47^

Due to their nutrient-rich composition, cocoa pulp and juice hold strong potential as functional ingredients in probiotic formulations, supporting both probiotic viability and the release of bioactive compounds.^47,48^ Analogous evidence from cocoa pulp juice supports its potential application in functional food formulations. In this regard, Tadtan et al.^49^ demonstrated the application of cocoa pulp juice, considered here as a related cocoa pulp side-stream, in a functional ice cream formulation composed of 95% pulp juice, 4% unfermented cocoa powder, and 1% carboxymethyl cellulose (CMC). The product exhibited desirable physicochemical properties, moderate sensory acceptability (scores 5.8–7.4), and ensured the viability of *Lactobacillus casei* subsp. *rhamnosus* during storage. Beyond maintaining probiotic activity, the formulation enhanced levels of bioactive compounds, including theobromine, catechin, and epicatechin, while also improving antioxidant activity, thereby reinforcing the potential of cocoa-based ingredients for the development of value-added functional products.

A recent study on the development of chocolate incorporating cocoa byproducts has attracted considerable attention. In this approach, a gel was formulated using cocoa husks combined with concentrated cocoa pulp juice, which was incorporated into the chocolate mass as a partial sugar substitute. It is important to note that although this pulp-derived ingredient may share some compositional similarities with cocoa honey, it is a distinct product due to differences in the extraction process. The resulting chocolate exhibited a sweeter taste compared to conventional formulations, along with compositional changes such as a higher fiber content and reduced saturated fatty acids; however, these differences should be interpreted within the specific formulation and experimental conditions described in the study without generalizing broader nutritional advantages.^48^

Patent-based evidence involving cocoa honey described the incorporation of cocoa honey in the production of brandy, outlining processing protocols that span from the harvesting of ripe fruits to the bottling of the final product. These documents present proposed methods, including fruit selection, washing and sanitization, extraction of pulp and/or cocoa honey, followed by enzymatic treatment, yeast inoculation, fermentation, and subsequent decantation and distillation. However, it is important to note that these patent descriptions represent claimed processes and do not necessarily constitute independently validated performance or product quality outcomes. Finally, the brandy undergoes stages of standardization, maturation, storage, and bottling, ensuring the product quality and consistency.^50^

The patent held by Rodrigues et al.^51^ describes the formulation of craft beer in which a portion of the must is replaced with cocoa honey, serving as a fermentable sugar source for yeast. According to the patent, the incorporation of cocoa honey is associated with characteristics such as a higher alcohol content, increased acidity, fruity aroma, and sweet flavor as well as the presence of flavonoids. However, it is important to note that these attributes are described within the patent as expected or claimed outcomes and are not necessarily supported by independently validated experimental data. Cocoa honey may be incorporated into beverages in both dehydrated and pasteurized forms.

As an alternative to commercial sugar, Nestlé published a patent in 2020 describing the use of dried cocoa pulp or cocoa honey in chocolate production. The patent proposes the use of these cocoa-derived ingredients as natural sources of sweetness, with the aim of reducing or replacing added sugar in the formulation. It also suggests potential impacts on flavor due to the retention of cocoa pod components. However, these aspects are presented as part of the patent’s proposed benefits and are not necessarily supported by independently validated comparative or sensory data.^52^

Direct evidence from cocoa honey suggests its potential application in cosmetic formulations. A startup, Cacaus Biocosmetics, has developed products such as a facial cream and a body lotion formulated with cocoa honey, aiming to explore its antioxidant, anti-inflammatory, and antimicrobial properties. Preliminary reports indicate an absence of skin irritation based on an in vitro/ex vivo model using the native human skin; however, the available information does not fully specify key methodological aspects such as the sample size, exposure duration, or evaluation criteria. Therefore, this claim should be interpreted with caution, and further well-designed studies, including human trials, are required to establish the safety and efficacy of cocoa honey in cosmetic applications.^53^

On the other hand, an invention explored the use of aromatic compounds derived from cocoa honey in the production of perfumes, colognes, and natural cocoa extracts. This approach highlights the potential of cocoa honey as a source of volatile compounds for applications in the fragrance industry; however, these applications are described within the context of a patent and should be interpreted as proposed uses rather than as validated outcomes.^54^

In this context, dos Santos de Oliveira et al.^55^ reported that patents for beverages produced from cocoa honey were primarily identified in Brazil based on their patent-mapping approach, reflecting efforts to reduce waste in chocolate production and promote more sustainable and efficient industrial processes. Complementing these findings, Nascimento et al.^5^ reported that approximately 80% of cocoa honey-related patents are Brazilian, highlighting the country’s leading role in this sector. To ensure reproducibility, the patent landscape analysis considered searches conducted between February 2024 and February 2025 using databases such as the National Institute of Industrial Property (INPI), Google Patents, Espacenet Patent Search, Patentscope (WIPO), and the Lens and Derwent Innovation, covering multiple jurisdictions. The search strategy included combinations of keywords such as “cocoa pod husk”, “cocoa pulp”, “cocoa honey”, and “cocoa bean shell”, applied to titles and abstracts.

Thus, the recognition of cocoa honey as a valuable ingredient aligns with a broader movement to sustainably utilize all components of the cocoa plant, contributing to the diversification of income for small producers and promoting more sustainable production practices.^48,56,57^

## 8 Limitations and Future Perspectives

Several research gaps remain regarding cocoa honey, including the lack of modern extraction equipment, the absence of standardized analytical methods, the scarcity of large-scale or long-term stability studies, limited assessments of economic feasibility, and insufficient data on sensory attributes and consumer acceptance. In addition, critical aspects for industrial adoption still need to be addressed, such as regulatory classification and labeling requirements (particularly regarding the use of the term “honey”), the establishment of microbial safety criteria and identification of HACCP relevant hazards, as well as supply chain and cold-chain constraints, including associated cost implications. Addressing these gaps requires comprehensive studies that provide robust technical, economic, and sensory evidence to support the development of cocoa honey as a safe, stable, and competitive product for both national and international markets.^5,6^

An additional critical limitation in the development of cocoa honey is the variability in its composition and microbiological stability. The physicochemical and nutritional profile of cocoa honey may vary significantly due to factors such as cocoa variety, seasonal conditions, harvest period, and postharvest handling practices, which can directly impact product quality and standardization.^58,59^ Moreover, its high moisture content and sugar-rich composition make it a favorable substrate for microbial growth, posing challenges for preservation and shelf life extension. These aspects highlight the need for further studies focused on understanding compositional variability and developing effective stabilization strategies to ensure product safety, consistency, and industrial applicability.^5,6,30^

The growing interest in cocoa honey as a nutritious and versatile ingredient opens up numerous opportunities for future research. Key areas include standardizing quality parameters, especially by identifying volatile organic compounds (VOCs) as authenticity markers, and developing new food and beverage applications such as natural sweeteners and functional ingredients. Research could also validate its health benefits such as antioxidant and anti-inflammatory properties.

Moreover, the identification of specific VOCs as potential quality markers may support future strategies for product differentiation and branding. However, the establishment of geographical indications or origin labels based on VOC profiles requires robust evidence of geographic discrimination, supported by validated data sets that distinguish the geographic origin from varietal and processing influences.^12,34^

From a sustainability perspective, integrating cocoa honey into circular economy models may contribute to waste reduction and promote more sustainable practices. However, its potential to improve farmers’ income remains a hypothesis that should be evaluated through techno-economic analyses and adoption studies.^60,61^ Additionally, its potential health benefits, including antioxidant and anti-inflammatory properties, should be investigated through a combination of in vitro assays, animal models, and well-designed human intervention studies to establish biological relevance and efficacy.

Cocoa honey represents a product with promising technological and innovation potential in both the food sector and other industries, such as cosmetics and pharmaceuticals. Its production may contribute to the sustainability and diversification of agricultural systems while offering opportunities for the development of value-added products. However, given the still limited body of evidence, particularly regarding its functional properties and large-scale applications, further studies are needed to better establish its potential benefits and industrial feasibility. In this context, this Review aims to support future research and technological developments that may improve the production, processing, and potential commercialization of cocoa honey.
